# Guidance Molecules in Vascular Smooth Muscle

**DOI:** 10.3389/fphys.2018.01311

**Published:** 2018-09-19

**Authors:** Alexandra Christine Finney, Anthony Wayne Orr

**Affiliations:** ^1^Department of Cellular Biology and Anatomy, Louisiana State University Health Sciences Center Shreveport, Shreveport, LA, United States; ^2^Department of Molecular and Cellular Physiology, Louisiana State University Health Sciences Center Shreveport, Shreveport, LA, United States; ^3^Department of Pathology and Translational Medicine, Louisiana State University Health Sciences Center Shreveport, Shreveport, LA, United States

**Keywords:** guidance molecules, vascular smooth muscle cells, pericytes, vascular remodeling, cardiovascular disease

## Abstract

Several highly conserved families of guidance molecules, including ephrins, Semaphorins, Netrins, and Slits, play conserved and distinct roles in tissue remodeling during tissue patterning and disease pathogenesis. Primarily, these guidance molecules function as either secreted or surface-bound ligands that interact with their receptors to activate a variety of downstream effects, including cell contractility, migration, adhesion, proliferation, and inflammation. Vascular smooth muscle cells, contractile cells comprising the medial layer of the vessel wall and deriving from the mural population, regulate vascular tone and blood pressure. While capillaries lack a medial layer of vascular smooth muscle, mural-derived pericytes contribute similarly to capillary tone to regulate blood flow in various tissues. Furthermore, pericyte coverage is critical in vascular development, as perturbations disrupt vascular permeability and viability. During cardiovascular disease, smooth muscle cells play a more dynamic role in which suppression of contractile markers, enhanced proliferation, and migration lead to the progression of aberrant vascular remodeling. Since many types of guidance molecules are expressed in vascular smooth muscle and pericytes, these may contribute to blood vessel formation and aberrant remodeling during vascular disease. While vascular development is a large focus of the existing literature, studies emerged to address post-developmental roles for guidance molecules in pathology and are of interest as novel therapeutic targets. In this review, we will discuss the roles of guidance molecules in vascular smooth muscle and pericyte function in development and disease.

## Introduction

Although both pericytes and vascular smooth muscle cells arise from the mural cell population and both contribute to vessel wall stability and tone (Kumar et al., 2017), these cells play critical but distinct roles in regulating vascular structure and remodeling. In smaller vessels and capillaries, pericytes cover and directly interact with the endothelial cell layer (Armulik et al., 2011). These interactions are crucial for vessel stability, as loss of pericyte coverage results in microvascular deficits such as hemorrhage or edema. This stabilization may be direct or indirect, as both endothelial cells and pericytes contribute to deposition of the subendothelial basement membrane (Stratman and Davis, 2012). In medium sized and large vessels, multiple layers of vascular smooth muscle and elastic fibers comprise the contractile medial layer of the vessel (Tucker and Bhimji, 2018). Vascular smooth muscle in arteries and veins primarily function to regulate vessel tone, but with the onset of vascular disease, such as atherosclerosis or vessel injury, smooth muscle cells shift to a pro-migratory and fibroproliferative phenotype (Owens et al., 2004). During this phenotypic modulation, smooth muscle cells downregulate their contractile markers, including smooth muscle α-actin (SMα-A), myosin heavy chain 11 (MHC11, or smooth muscle myosin heavy chain), Calponin, and Leiomodin, and upregulate the expression of genes involved in extracellular matrix deposition and inflammation (Owens et al., 2004). The accumulation of vascular smooth muscle in atherosclerotic or injured vessels results in luminal narrowing, thereby reducing blood flow, but also in the formation of a fibrous cap that prevents exposure of blood to the thrombogenic necrotic core of the plaque (Chamie et al., 2011; Marx et al., 2011). Therefore, therapeutics targeting vascular smooth muscle proliferation and migration should be carefully considered given these opposing outcomes.

**FIGURE 1:**
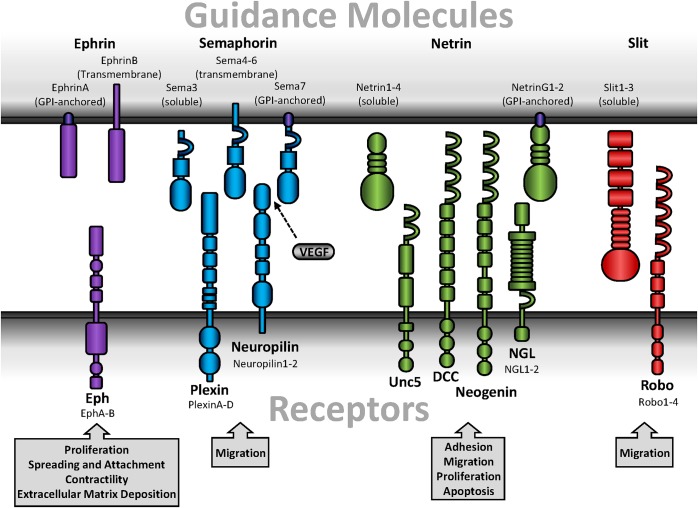
Schematic diagrams of guidance molecules. Guidance molecules include ephrins, semaphorins, netrins, and Slits. Ephrins interact with Eph receptors and are designated as type A or B based on sequence homology, binding affinity, and ligand anchorage. Semaphorins are either bound or secreted and interact with either plexins or neuropilins. Neuropilin, while a receptor for guidance molecules, additionally interact with several forms of VEGF. Secreted netrins interact with Unc, DCC, or neogenin receptors, while GPI-anchored netrins interact with NGL. Slits are secreted but proteolytically processed into a short, non-functional fragment and a long, functional fragment that remains associated with the cell membrane and interacts with Robo.

Guidance molecules play well-described roles in neuronal development and tissue patterning. The four major guidance molecules are highly conserved amongst species and consist of ephrins, semaphorins, netrins, and Slits (**Figure 1**). These guidance molecules can either be secreted or function as cell surface-bound ligands that interact with their cognate receptors on neighboring cells. In general, guidance molecules promote attractive or repulsive cues through alterations in Rho family GTPase (Rho, Rac, Cdc42) activation which regulate cytoskeletal dynamics (Fritz and VanBerkum, 2002). While Rac and Cdc42 promote actin polymerization and focal adhesion turnover at the leading edge of a cell to facilitate migration, Rho stimulates actinomyosin-mediated contractility and focal adhesion assembly which promotes cell rounding and retraction (Raftopoulou and Hall, 2004). Initially characterized with the search for axonal chemoattractants and studies of guidance defects (Dickson, 2002), guidance molecules have emerged as potent regulators in post-developmental response such as angiogenesis or injury-induced neuronal plasticity (Eichmann et al., 2005; Adams and Eichmann, 2010; Hollis, 2016). While studies primarily identified post-developmental contributions for guidance molecules in endothelial cells (Adams and Eichmann, 2010) and leukocyte-mediated inflammation (Ranganathan et al., 2014; Chaturvedi and Robinson, 2015; Vadasz et al., 2015), less is explored in vascular smooth muscle biology.

Ephrin ligands bind to Eph receptors (**Figure 1**), the largest mammalian subfamily of receptor tyrosine kinases in the mammalian genome. Eph receptors are designated as subtypes A and B due to sequence homology and binding preferences (Flanagan et al., 1997). Ephrins (Eph receptor interacting proteins) are similarly defined as either type A or B based on membrane anchorage and receptor binding. EphAs primarily interact with GPI-anchored ephrinAs, whereas EphBs primarily interact with transmembrane ephrinBs. However, there are a few exceptions of receptor-binding promiscuity (Smith et al., 1997). In general, expression patterns of each subtype determine receptor-ligand interactions. In vascular smooth muscle cells, EphA2, EphA4, EphA5, and EphA7 are highly expressed in conjunction with ephrinA4 and ephrinA5 (Finney et al., 2017). Additionally, EphB4 and EphB6 are the predominant EphB receptors in smooth muscle, and are co-expressed with ephrinB1, ephrinB2, and ephrinB3 (Kudo et al., 2007; Luo et al., 2012).

Upon ligation, signaling can occur on the Eph receptor-expressing cell (forward signaling) or the ephrin ligand-expressing cell (reverse signaling). In general, ligation promotes clustering of the Eph receptors and allows for transphosphorylation and kinase activation. Termination of signaling occurs through Eph and ephrin proteolytic processing (e.g., ADAM metalloproteinases, γ-secretase) leading to internalization and degradation (Fasen et al., 2008; Boissier et al., 2013; Atapattu et al., 2014). However, proteolytic processing sheds soluble fragments and has the potential to interact with non-adjacent cells, therefore serving as a useful biomarker for disease (Barquilla and Pasquale, 2015). Eph/ephrin signaling affects a variety of downstream signaling pathways to influence cytoskeletal remodeling [RhoA, focal adhesion kinase (FAK)] or proliferation [Akt, extracellular regulated kinase (ERK)] (Pandey et al., 1994; Miao et al., 2000; Deroanne et al., 2003; Ogita et al., 2003; Menges and McCance, 2008). Classic Eph receptor forward signaling inhibits migration and promotes cell rounding, which facilitate tissue boundary formation during development (Cayuso et al., 2015). Intriguingly, recent studies identified a novel role for EphA2 ligand-independent signaling in which non-ligated EphA2 promotes proliferation, migration, and invasion independent of its kinase activity (Miao et al., 2009).

Semaphorins are a large family of both secreted and membrane-bound glycoproteins divided into eight classes (Classes 1–7, V) based on structure and sequence homology (Goodman et al., 1999). First described in *Drosophila* as an axonal guidance cue in the developing central and peripheral nervous systems (Kolodkin et al., 1993), Semaphorins mediate their function through two types of receptors, plexins (Classes A–D) and Neuropilins (Nrp1 and 2) (**Figure 1**) (Kameyama et al., 1996; He and Tessier-Lavigne, 1997). Vertebrates only express Sema3-7, and are further divided into a total of 19 subgroups (Goodman et al., 1999). Class 3 Semaphorins (Sema3A-G) are secreted. In contrast, class 4–6 Semaphorins (Sema4A-G, Sema5A-B, Sema6A-D) are transmembrane, while the class 7 Semaphorin (Sema7A) is GPI-anchored (Goodman et al., 1999; Koncina et al., 2007). Associations with Neuropilins or plexins differ between the Semaphorin subgroups to give rise to selective cellular responses (**Table 1**). For example, Sema3A binds Neuropilin1, plexinA1-4, plexinB1, plexinD1, and neural adhesion molecules (Sharma et al., 2012). Sema4D and Sema7A, however, bind plexin-B1 and plexin-C1, respectively (Pascoe et al., 2015). While Sema3C was the only published Semaphorin expressed by vascular smooth muscle for some time (Lepore et al., 2006), recent proteomic analysis suggests multiple Semaphorins are detected in smooth muscle (Uhlen et al., 2015). However, Semaphorins expressed by other cell types have been shown to facilitate signaling through plexin or Neuropilin receptors on vascular smooth muscle. Indeed, vascular smooth muscle cells express both Nrp1 and Nrp2, as well as plexin-D1 (Bielenberg et al., 2012; Movassagh et al., 2014; Yamaji et al., 2015), all of which have the capacity to bind with Semaphorins.

**Table 1:** Summary of semaphorin family interactions.

Receptor	Ligand(s)	Reference
PlexinA1	Sema2C, Sema3A, Sema3F, Sema3G, Sema5A, Sema5B, Sema6A, Sema6C, Sema6D	Yoshida et al., 2006; Mauti et al., 2007; Matsuoka et al., 2011; Hayashi et al., 2012; Delloye-Bourgeois et al., 2015; Uchida et al., 2015
PlexinA2	Sema3A, Sema3B, Sema3C, Sema3F, Sema3G, Sema5A, Sema6A, Sema6B	Takahashi and Strittmatter, 2001; Suto et al., 2007; Kodo et al., 2009; Andermatt et al., 2014; Duan et al., 2014; Sabag et al., 2014; Uchida et al., 2015
PlexinA3	Sema3A, Sema3D, Sema3F, Sema5A, Sema5AB	Bagri et al., 2003; Yaron et al., 2005; Matsuoka et al., 2011; Ton and Kathryn Iovine, 2012
PlexinA4	Sema3A, Sema3B, Sema6A, Sema6B	Suto et al., 2005, 2007; Sabag et al., 2014
PlexinB1	Sema4A, Sema4D	Tamagnone et al., 1999; Yukawa et al., 2010
PlexinB2	Sema4A, Sema4C, Sema4G	Yukawa et al., 2010; Maier et al., 2011; Gurrapu et al., 2018
PlexinB3	Sema4A, Sema5A	Artigiani et al., 2004; Yukawa et al., 2010
PlexinC1	Sema7A	Tamagnone et al., 1999
PlexinD1	Sema3A, Sema3C, Sema3D, Sema3E, Sema3G, Sema4A	Gitler et al., 2004; Chauvet et al., 2007; Toyofuku et al., 2007; Hamm et al., 2016; Liu X. et al., 2016
Neuropilin1	Sema3A, Sema3B, Sema3C, Sema3D, Sema3F, VEGF-A, VEGF-B, VEGF_165_	Chen et al., 1997; Soker et al., 1998; Long et al., 2009; Hayashi et al., 2012; Sabag et al., 2014; Jensen et al., 2015; Hamm et al., 2016
Neuropilin2	Sema3C, Sema3B, Sema3D, Sema3F, Sema3G, VEGF_121_, VEGF_165_	Chen et al., 1997; Gluzman-Poltorak et al., 2001; Ton and Kathryn Iovine, 2012; Sabag et al., 2014; Uchida et al., 2015
CD72	Sema4D	Kumanogoh et al., 2000
TIM-2	Sema4A	Kumanogoh et al., 2002
α1β1 integrin	Sema7A	Suzuki et al., 2007
L1CAM	Sema3A	Castellani et al., 2000

*The diverse family of semaphorin guidance molecules are designated as Semaphorin3(A-G), Sema4(A-F,G), Sema5(A-B), Sema6(A-D), and Sema7(A). This comprehensive table provides all currently known interactions between semaphorins and their receptors*.

Netrins exhibit structural similarities with the laminin family (Serafini et al., 1994), and act as either an attractive or repulsive stimulus mediated by specific receptor interactions. Five mammalian netrin subtypes are divided into either secreted ligands (netrin-1, netrin-3/Netrin-2-like, netrin-4) or GPI-anchored ligands (netrin-G1 and netrin-G2) (Moore et al., 2007). While Netrin-3 is expressed in mice, the human homolog is Netrin-2-like, named for its similarities with chick Netrin-2 (Serafini et al., 1994). Furthermore, vascular smooth muscle cells only express netrin-1 and netrin-4 (Brunet et al., 2014; Lejmi et al., 2014). Secreted netrins bind DCC, neogenin, and Unc5, which are differentially expressed in various cell types but all expressed in vascular smooth muscle (Ly et al., 2005; Moore et al., 2007; Lejmi et al., 2014). In contrast, netrin-G1 and netrin-G2 bind netrin-G ligands (NGL-1 and NGL-2, respectively) and are primarily expressed in the central nervous system (**Figure 1**) (Moore et al., 2007; Seiradake et al., 2011). In general, netrin receptor activation promotes reorganization of the actin cytoskeleton, which contributes to cell motility (Duman-Scheel, 2009), although other pathways have been implicated with netrin signaling (Hong et al., 2000; Ziel and Sherwood, 2010). While binding to DCC or neogenin promotes chemoattraction, ligation of netrin to Unc5 induces chemorepulsion (Oksala et al., 2013).

Originally identified in *Drosophila*, Slit ligands function through interactions with their cognate receptor Roundabout (Robo) (**Figure 1**) (Seeger et al., 1993; Brose et al., 1999). Full-length Slit ligands are secreted but remain closely associated with the cell membrane (Brose et al., 1999), where they are proteolytically processed into a short C- and long N-terminal fragment (Brose et al., 1999). Although the *Drosophila* homolog to pheromone convertase was recently identified as a Slit protease (Ordan and Volk, 2016), the vertebrate protease remains unknown. While full-length and N-terminal Slit ligands are capable of binding Robo, the C-terminal fragment is inactive (Nguyen Ba-Charvet et al., 2001), suggesting proteolytic processing is a crucial regulator of Slit–Robo signaling. Vertebrates express four different Robo receptors (Robo1-4), and three different Slit ligands (Slit1-3). While all isoforms are highly expressed in the central and peripheral nervous systems (Carr et al., 2017), only Slit2, Slit3, Robo1, Robo2, and Robo4 are expressed in rat carotid arteries (Liu D. et al., 2016). Classically, Slit–Robo interactions provide a repulsive cue to maintain midline deviation of neuronal axons in CNS development (Dickinson and Duncan, 2010; Barak et al., 2014). Consistent with this, interactions between Robo1 and Slit2 mediates detachment and chemorepulsion of diabetic T-cells on endothelial cells *in vitro* (Glawe et al., 2013), implicating a role for Slit–Robo signaling in cardiovascular disease. Somewhat unique to Slit–Robo interactions, extracellular matrix-immobilized Slit binds Robo which creates cellular tension and thus exposes a protease binding site on Robo (Barak et al., 2014). The Robo receptor is proteolytically processed by ADAM family metalloproteinases during CNS midline development in *Drosophila* (Coleman et al., 2010), which facilitates repulsion away from initial contact with the Slit ligand Although the Robo metalloproteinase cleavage site is conserved between *Drosophila* and humans (Seki et al., 2010), a specific function in human Robo proteolytic processing remains unaddressed.

In this review we will address how guidance molecules influence pericyte and smooth muscle incorporation during vascular development and remodeling with a focus on how these guidance cues regulate migration and proliferation of the mural cell population. We will discuss how these guidance cues coordinate vascular patterning between blood vessels and peripheral nerves and address the paracrine effects these molecules have on crosstalk between neurons and vascular smooth muscle cells. Post-developmentally, we summarize how guidance molecules contribute to cardiovascular disease through alterations in blood pressure regulation, atherosclerotic plaque formation, and restenosis vascular remodeling. Lastly we will address how these various guidance molecules provide potential therapeutic targets in cardiovascular disease.

## Vascular Patterning and Innervation

The role of guidance molecules in vascular patterning has been summarized previously with emphasis on endothelial cell biology (Adams and Eichmann, 2010). However, mural cells also play key roles in vascular development. A functional vascular network is crucial for survival during embryogenesis and subsequent organ development (Wang et al., 2015a). The effects of guidance molecules on vascular patterning are well-described, as repulsive cues between tissue types designate organ boundaries upon ligand-receptor ligation. Given their retractive nature upon interaction, EphB4 and ephrinB2 are crucial for designating vascular tissue patterning in development and classically define venous and arterial identity, respectively (Kudo et al., 2007). Indeed, deletion of either protein results in embryonic lethality due to cardiovascular defects (Gerety et al., 1999). While direct cell-to-cell contact commonly activates guidance molecules signaling, soluble ligands have the potential to effect both neighboring and distant cells. For example, vascular smooth muscle secretes Slit3 to regulate migration of endothelial cells or other vascular smooth muscle cells, and treatment with Slit3 promotes neovascular formation (Zhang et al., 2009). Furthermore, vascular smooth muscle secretes netrin-1 to promote sympathetic innervation of resistance arteries (Brunet et al., 2014).

Development of the cardiovascular system is critical for survival and thus occurs early in organogenesis. First involving differentiation of mesoderm-derived angioblasts with vascular endothelial growth factor (VEGF) stimulation, endothelial cells assemble into *de novo* functional vessels, termed vasculogenesis (Udan et al., 2013). To promote stability and functionality of newly formed vasculature, mural cells are recruited and differentiate into either vascular smooth muscle (in veins/arteries) or pericytes (in capillaries) (Wang et al., 2015a). The origin of vascular smooth muscle and pericytes is diverse, as they derive from multiple embryonic sources including the mesoderm and ectoderm (Pfaltzgraff and Bader, 2015). These differential origins of vascular smooth muscle contribute to distinct vascular beds. For example, smooth muscle derived from the neural crest gives rise to the aortic arch, ascending aorta, brachiocephalic trunk, and common carotid arteries. In contrast, vascular smooth muscle in coronary arteries arise from the proepicardium (Majesky, 2007). Despite this, local recruitment to early vascular structures involve differentiation of the mural cells into smooth muscle or pericytes, migration toward blood vessels, and proliferation in the developing vascular wall (Hellstrom et al., 1999; Goumans and Ten Dijke, 2018).

**FIGURE 2:**
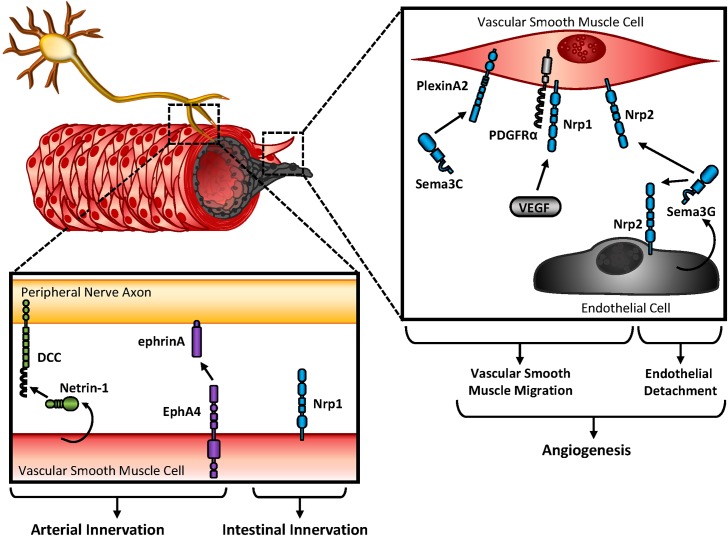
Vascular patterning and innervation. Peripheral nerve axons and endothelial cells rely on crosstalk with vascular smooth muscle cells during angiogenesis, and innervation. Either secreted or bound guidance molecules stimulate their cognate receptors to promote migration and attachment during development.

Innervation of the vasculature by the autonomic nervous system provides regulation of both vessel contraction and arterial differentiation (Mukouyama et al., 2002; Eichmann and Brunet, 2014). These responses are regulated by either substrate or cellular-bound stimuli (extracellular matrix, membrane-bound ligands or receptors) (Gale and Yancopoulos, 1999; Sottile, 2004; Lejmi et al., 2014) or secreted soluble factors (growth factors, guidance cues) (Hellstrom et al., 1999; Kutschera et al., 2011; Goumans and Ten Dijke, 2018). Since secreted ligands exhibit the capability of affecting multiple cell types in the vessel wall (endothelium, vascular smooth muscle/pericytes, nerves), differential expression of their cognate receptors is crucial for a targeted response. For example, the secreted Semaphorin, Sema3C, promotes neural crest-derived smooth muscle migration through interactions with smooth muscle’s plexinA2 receptor (**Figure 2**) (Lepore et al., 2006). Consistent with this, Sema3A’s receptor Nrp1 localizes with platelet-derived growth factor (PDGF) receptor α (PDGFRα) on vascular smooth muscle cells to promote migration and p130^Cas^-mediated chemotaxis (Pellet-Many et al., 2011). While not required for vascular development, smooth muscle-expressed Nrp1 also interacts with VEGF family members to promote angiogenesis in endothelial cells. Since Sema3G is expressed in endothelial cells but essentially undetected in vascular smooth muscle, endothelial-derived Sema3G may have paracrine effects on vascular smooth muscle or endothelial function (**Figure 2**) (Kutschera et al., 2011). Although smooth muscle and endothelial cells express Nrp2, Sema3G exerts differential responses on each cell type. While Sema3G promotes smooth muscle migration, exogenous Sema3G rapidly induces endothelial cell detachment from coculture with smooth muscle, suggesting Sema3G signaling leads to vascular destabilization (Kutschera et al., 2011).

Guidance cues promote innervation of developing blood vessels, and additionally contribute to reinnervation of the vessel wall post-injury. While smooth muscle contractility is regulated by hemodynamics, endothelial cues, and circulating factors, blood vessel innervation by sympathetic or parasympathetic nerves also regulates contractility through the release of neurotransmitters. While sympathetic nerves release norepinephrine and promote vasoconstriction through vascular smooth muscle contraction, parasympathetic nerves release acetylcholine and promote vasodilation through nitric oxide (NO) release by the endothelium (Amiya et al., 2014), During cardiovascular development, arteries and sympathetic nerves align prior to direct innervation of the vascular wall, which may be explained by similar responsiveness to spatial and temporal expression of guidance cues. Large compliance arteries utilize their elasticity to buffer large fluctuation in blood pressure during the cardiac cycle, and are minimally innervated. In contrast, muscular arteries and arterioles show abundant innervation and largely contribute to vasoconstriction (Storkebaum and Carmeliet, 2011). Parallel to this observation, Netrin-1 shows minimal expression in large elastic arteries in contrast to muscular arteries, where Netrin-1 is readily detectable (Brunet et al., 2014). In addition, Netrin-1’s cognate receptor, DCC, is highly expressed in sympathetic nerves, suggesting the interactions between cell types may mediate vascular innervation (**Figure 2**). Indeed, deletion of Netrin-1 from vascular smooth muscle or blocking DCC receptors reduces arterial innervation (Brunet et al., 2014).

Ephs and ephrins are expressed on both nerve fibers and vascular smooth muscle. Following denervation of the femoral artery, soluble EphA4 blunts reinnervation (**Figure 2**) (Damon et al., 2010). Since soluble EphA4 treatment likely affects multiple cell types, it is unclear whether EphA4-inhibited reinnervation is due to effects on peripheral nerve or vascular smooth muscle function. Soluble EphA receptors either (a) activate reverse signaling on the peripheral nerve, since ephrinA interacts with neurotrophin receptors to enable repulsive cues in retinal axons (Marler et al., 2010) or (b) block forward signaling on the vascular smooth muscle. Experimental models would favor the latter scenario, since EphA2 expression is required for fibronectin deposition (Hu et al., 2004; Finney et al., 2017) and fibronectin is hypothesized to guide peripheral nerves on vascular smooth muscle (Spence and Poole, 1994). Furthermore, soluble EphA4 enhances norepinephrine release by the peripheral nerve without affecting norepinephrine uptake by the vascular smooth muscle, suggesting EphA4 treatment does affect peripheral nerve function during reinnervation (Damon et al., 2010).

Semaphorin3A regulates peripheral nerve guidance by binding with Neuropilin1 (Bates et al., 2003), and global deletion of Neuropilin1 leads to vascular defects and embryonic lethality (Yamaji et al., 2015). Although vascular smooth muscle provides tropic cues for peripheral axons during vascular innervation, conditional deletion of Neuropilin1 shows no overt cardiovascular abnormalities (Yamaji et al., 2015). However, deletion of smooth muscle Neuropilin1 blunts intestinal innervation and contractility (Yamaji et al., 2015), suggesting smooth muscle guidance cues also regulate innervation of other organ systems (**Figure 2**).

## Pericyte Recruitment to Stabilize Angiogenic Vessels

Pericytes and vascular smooth muscle cells exhibit parallel functions in contractility and endothelial cell coverage, although pericytes are found in the microvasculature. Similar to vascular smooth muscle cells, pericytes are derived from multiple germ cell layers but display a variety of functions depending on the anatomical location of the vascular bed. During embryogenesis, pericytes are recruited to developing vascular beds by endothelial-derived soluble factors, and direct interactions with the endothelium regulate mural cell proliferation and migration (Davis et al., 2011). In the central nervous system, pericytes contribute to the neurovascular unit, signaling with neighboring endothelial cells, neurons, and glia to regulate capillary diameter, vascular stability, and leukocyte immune response (Winkler et al., 2011). While endothelial cells are well-established for their roles in vascular function, pericytes’ diverse contributions to the vessel wall are becoming increasingly appreciated (Armulik et al., 2005). Indeed, pericyte loss or dysfunction results in disruption of the endothelial barrier, leading to a myriad of pathologies including diabetic retinopathy, hemorrhage, and tumor metastasis (Ferland-McCollough et al., 2017).

Eph and ephrin signaling is a known mediator of angiogenesis both developmentally and in tumors, which highly express tumor and vascular EphA2 (Brantley-Sieders et al., 2004; Lin et al., 2007; Sainz-Jaspeado et al., 2013). Although these studies primarily focus on endothelial EphA2, mural cells are still influenced by Eph-ephrin signaling through crosstalk with the endothelium. *In vitro*, EphA2 upregulation is observed with differentiation of an embryonic cell line to pericytes, suggesting a potential role for EphA2 in pericyte development (Kale et al., 2005). Indeed, deletion of EphA2 reduces tracheal pericyte coverage of young mice with enhanced capillary diameter. Intriguingly, these deficits were lost as mice aged, suggesting a compensatory response post-development (Okazaki et al., 2009). Although EphA2 expression promotes smooth muscle cell proliferation which could explain the defect in pericyte recruitment (Finney et al., 2017), no studies to date directly link EphA2-mediated proliferation in pericyte recruitment.

**FIGURE 3:**
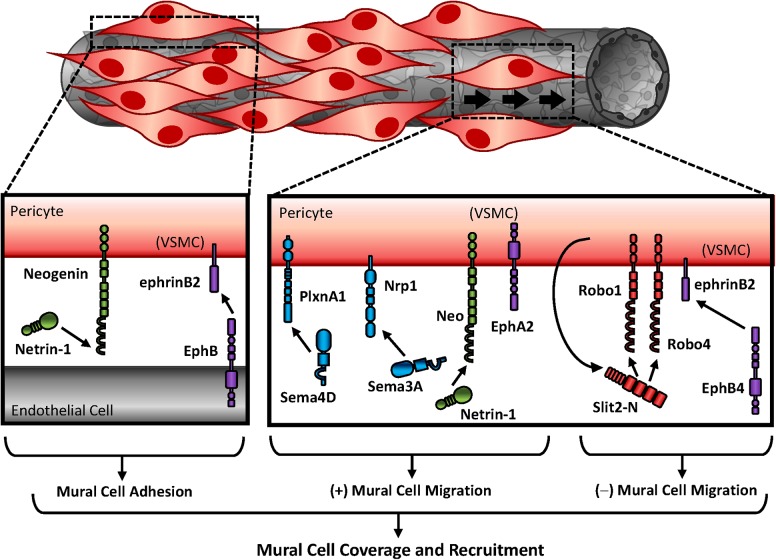
Mural cell recruitment to stabilize angiogenic vessels. Differential expression of guidance molecules regulates cellular response. While semaphorins and netrins promote mural cell migration, Slits and ephrins inhibit mural migration. Studies that utilized vascular smooth muscle cells for *in vitro* experiments are indicated as “(VSMC).”

Proliferative influences by Ephs and ephrins may be unique to the EphA subfamily, as deletion of ephrinB or EphB family members show no alterations in vascular smooth muscle proliferation (Wang et al., 2016a). In contrast, EphB-ephrinB signaling predominantly regulates cell adhesion to modulate pericyte recruitment and vascular wall assembly (**Figure 3**). LacZ reporter labeling of ephrinB2 showed high expression in vascular smooth muscle and pericytes, although this expression was not evident early in development (Gale et al., 2001). Despite this, ephrinB2 deletion was embryonic lethal with severe hemorrhaging and edema indicative of vascular wall deficits. Upon closer inspection, ephrinB2 deletion from the mural cell population resulted in reduced pericyte coverage but did not alter pericyte numbers, suggesting ephrinB2 is crucial for pericyte spreading and attachment to the endothelium rather than proliferation (**Figure 3**) (Foo et al., 2006). Consistent with this, blockade of ephrinB2 reduces pericyte recruitment to Kaposi sarcoma tumors (Scehnet et al., 2009), and degradation of ephrinB2 phenocopies ephrinB2 knockout mice (Foo et al., 2006). Although EphB4-ephrinB2 interactions inhibit migration, ephrinB2 is required for focal adhesion maturation (**Figure 3**) (Foo et al., 2006; Salvucci et al., 2009). Therefore, ephrinB2 signaling likely promotes pericyte recruitment through alterations in cellular adhesion rather than proliferation.

While recruitment of pericytes requires cell adhesion to the endothelial layer, endothelial cells additionally influence pericytes through soluble factor release. In cancer biology, tumor cells secrete Sema4D to promote PDGF-B release, although the specific source is unclear, to enhance pericyte association with endothelial cells. In contrast, silencing Sema’s cognate receptor PlexinB1 inhibits pericyte migration (**Figure 3**) (Zhou et al., 2014). Similar to PlexinB1 deletion, blockade of Sema’s other cognate receptor Nrp1 reduces smooth muscle and pericyte coverage (Jurisic et al., 2012), and Nrp1 knockout mice are embryonic lethal due to cardiovascular defects. While Neuropilin1 function is essential in endothelial motility and growth cone collapse, the specific contributions in mural cells is less known (Yamaji et al., 2015). Sema3A interactions with Nrp1 promote pericyte migration (**Figure 3**) (Jurisic et al., 2012). Furthermore, exogenous Sema3A enhances pericyte coverage to normalize tumor vasculature, which promotes normoxia and chemotherapeutic delivery (Maione et al., 2009). The migratory effects of Sema3A appear to be receptor-specific, however, as interactions with plexinD1 inhibit migration of vascular smooth muscle cells through alterations in the actin cytoskeleton (Movassagh et al., 2014). Together these data suggest differential expression of the Semaphorin receptors determine cell response.

The effects of guidance molecule signaling in mural cells is also observed in brain microvasculature. Forced overexpression of Netrin-1 with an adeno-associated virus largely targets neurons and astrocytes in the brain. Since Netrin-1 is secreted, however, the effects of overexpression may be observed in neighboring cells. Consistent with this, Netrin-1 overexpression augments vessel diameter (Fan et al., 2008), suggesting Netrin-1-expressing neurons and astrocytes influence vascular morphology. While forced overexpression of netrin1 does not enhance brain microvessel quantity, Netrin-1 enhances proliferation of smooth muscle actin-positive regions *in vivo* while promoting proliferative and migratory responses of vascular smooth muscle cells *in vitro* (**Figure 3**) (Fan et al., 2008). These effects appear to be through interactions with neogenin, since DCC and Unc5H2 are undetected in human vascular smooth muscle cells and blockade of neogenin inhibits smooth muscle adhesion and migration in culture (Park et al., 2004). Intriguingly, soluble Netrin-1 does not affect migration or proliferation of non-vascular or endothelial cells, despite endothelial expression of neogenin (Lejmi et al., 2014). Furthermore, Netrin-4 paradoxically inhibits VEGF-induced endothelial migration and angiogenesis (Lejmi et al., 2008). These responses may be due to interactions with Netrin’s other cognate receptors on the endothelium, as endothelial Netrin-4 utilizes the recruitment of Unc5B with neogenin, which are both required for endothelial signaling (Larrivee et al., 2007).

Not much is known about Slit/Robo signaling in smooth muscle and pericyte recruitment. While the receptors Robo1 and Robo4 are expressed on endothelial cells and smooth muscle cells, Slit2 is only expressed in vascular smooth muscle. Slit2 proteolytic cleavage produces a small fragment (Slit2-C), whose function is minimally understood, and a larger fragment (Slit2-N), which remains tightly bound to the membrane and interacts with Robo receptors (Brose et al., 1999). While recent studies identified PlexinA1 as a potential receptor for Slit2-C in axonal guidance (Delloye-Bourgeois et al., 2015), Slit2-N inhibits smooth muscle migration through inhibition of Rac1 (Liu et al., 2006). Consistent with this, Slit2 inhibits pericyte migration through interactions with Robo1 and Robo4 and implies Slit–Robo signaling inhibits pericyte recruitment during angiogenesis (**Figure 3**) (Guijarro-Munoz et al., 2012).

## Blood Pressure Regulation and Contractility

**FIGURE 4:**
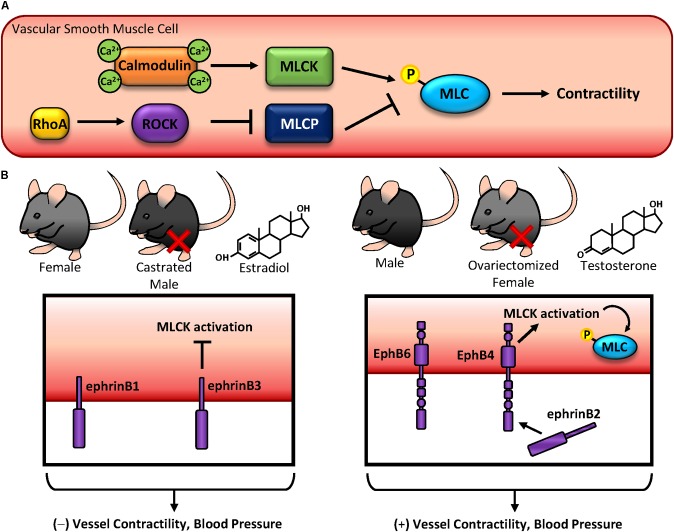
Blood pressure regulation and contractility. **(A)** Vascular smooth muscle contractility is regulated by phosphorylation of myosin light chain (MLC). MLC can become phosphorylated by either calcium-dependent calmodulin-MLC kinase, or by RhoA-ROCK-MLC phosphatase. **(B)** EphB and ephrinB expression on vascular smooth muscle regulates contractility and blood pressure in a sex hormone-specific manner. While ephrinB expression in females, castrated males, and estradiol-treated vascular smooth muscle cells inhibit contractility, EphB expression in males, ovariectomized females, and testosterone-treated vascular smooth muscle cells produces the opposite effect.

Contractile responses in vascular smooth muscle cells are tightly regulated by several factors: (1) neurotransmitter release from the neuromuscular junction signals for alterations in contractility (2) Local vasodilator release (NO, prostacyclin) from endothelial cells promotes vasodilation (3) Distal release of angiotensin from the suprarenal glands promotes vasoconstriction and (4) Alterations in pressure or vascular stiffness promotes signaling through mechanotransduction. Upon stimulation with a vasoconstrictor (either through neural or endocrine sources), intracellular calcium is released from the sarcoplasmic reticulum through IP3 signaling. Calcium binds with calmodulin to activate myosin light chain (MLC) kinase, which phosphorylates MLC to promote crossbridge formation and subsequent contraction (**Figure 4A**) (Mizuno et al., 2008). In contrast, MLC can be activated independent of calcium through RhoA-Rho kinase (ROCK) signaling, inhibiting MLC phosphatase and enhancing MLC phosphorylation and contractility (**Figure 4A**) (Amano et al., 1996). Perturbations in these cellular processes lead to cytoskeletal remodeling and stiffening, reducing vascular compliancy and manifesting as hypertension. While multiple factors exacerbate hypertension, like age, obesity, and smoking (Touyz et al., 2018), gender differences are well established when factoring risk for hypertension. Consistent with other cardiovascular diseases, men are at a higher risk for developing hypertension than women (Sandberg and Ji, 2012), although the mechanisms behind this are not well understood.

Since Eph-ephrin signaling alters cytoskeletal dynamics, these changes also have the potential to regulate contractility in vascular smooth muscle cells. While no studies to date identify a role for EphA-ephrinA signaling in blood pressure, Ogita et al. (2003) showed EphA4 promotes RhoA activation through ephrinA1-mediated activation of VsmRhoGEF which acts to promote MLC activation. However, these effects are unclear, as Deroanne et al. (2003) showed ephrinA1 treatment does not activate RhoA, although ROCK-mediated spreading was inhibited. These conflicting data may be due to ephrinA1 interactions with EphA4 versus EphA2, although neither group examined potential contributions from both receptors. In contrast with EphA signaling, ephB/ephrinB SNPs have been associated with blood pressure phenotypes (Wang et al., 2016b). Since multiple EphBs and ephrinBs are highly expressed in vascular smooth muscle (Luo et al., 2012) and early studies identified key roles in Eph-mediated contractility (Deroanne et al., 2003; Ogita et al., 2003; Wang et al., 2016c), these implicate Ephs and ephrins as potential drivers of blood pressure regulation (**Figure 4B**). Intriguingly, many of these findings appear to be sex hormone-specific, as differential effects are observed between males and females and following castration or ovariectomy (Luo et al., 2012; Wang et al., 2016c), suggesting a previously unidentified link between Eph-ephrins and sex hormone signaling.

EphrinB1 reverse signaling promotes tyrosine phosphorylation to enhance interactions with GRIP1, a transcriptional co-activator of steroid hormone receptor (Hong et al., 1997). Although GRIP1 has been shown to interact with RhoGDI (Su et al., 2001), and the interaction of EphrinB1 with GRIP1 abrogates RhoA activation and contractility, a specific mechanism by which this occurs is unclear (Wu et al., 2012; Wang et al., 2015c). Despite this, deletion of ephrinB1 enhances blood pressure (**Figure 4B**) (Wang et al., 2016a). Similarly, ephrinB3 deletion also enhances blood pressure, although this response is only observed in females and castrated males. Consistent with this, ovariectomy reverses this observation in females. These increases in blood pressure may be due to alterations in contractility, as exogenous estradiol treatment of ephrinB3-deleted cells enhances contractility through the G protein-coupled estrogen receptor (GPER), a G protein-associated surface molecule (Wang et al., 2016c). In contrast to estrogen-associated responses in ephrinB signaling, the effects of testosterone and smooth muscle contractility depend on both EphB and ephrinB expression. While testosterone or ovariectomy reduces contractility of ephrinB3 knockout cells, male EphB6 knockout mice also show reduced mesenteric artery contraction (**Figure 4B**) (Luo et al., 2012; Wang et al., 2016c). Despite these observations, testosterone may only affect reverse signaling, as cytoplasmic deletion of EphB6 does not alter blood pressure in male mice (Luo et al., 2012). Although this redundancy in ephrinB reverse signaling suggests crucial biological functions in vascular smooth muscle contractile response, other EphB receptors do regulate cell contractility and blood pressure through forward signaling. Upon ligation with ephrinB2, the EphB4 kinase domain becomes activated leading to enhanced contractility through alterations of MLC phosphorylation (**Figure 4B**) (Wang et al., 2016a). Consistent with this, deletion of EphB4 or inhibition of EphB4 kinase activity leads to hypotension and reduced MLC kinase activity. While calcium is a well-established effector of MLC-mediated cell contractility (Amberg and Navedo, 2013), the changes in smooth muscle contraction observed with EphB-ephrinB modulation are independent of calcium (Wang et al., 2015c, 2016a, 2016c). Again, these effects appear to be sex specific, as reduced blood pressure was only observed in male EphB4 knockout mice but not female knockout mice (Wang et al., 2015c). Furthermore, alterations in EphB/ephrinB expression do not affect endothelial-mediated vascular smooth muscle contraction, as denudation of the endothelium *in vitro* does not alter EphB-ephrinB-mediated vascular smooth muscle contractility (Wu et al., 2012; Wang et al., 2015c).

## Smooth Muscle Contributions to Atherosclerosis and Restenosis

**FIGURE 5:**
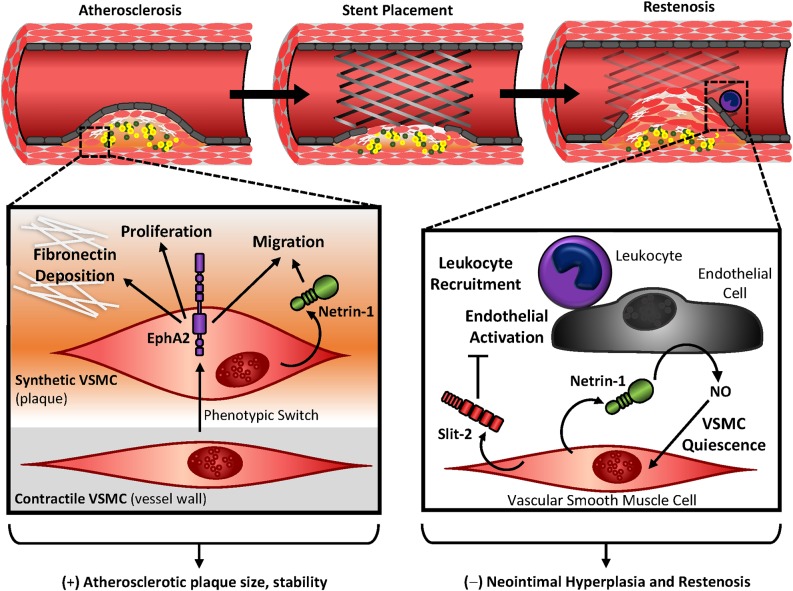
Vascular smooth muscle contributions in atherosclerosis and restenosis. During atherosclerotic development or vascular injury, vascular smooth muscle cells shift to a proliferative and migratory phenotype, where they accumulate in the neointima and deposit extracellular matrix proteins. In atherosclerosis, the abundance of vascular smooth muscle contributes to plaque size but confers protection with the formation of a fibrous cap. EphA2 expression in synthetic smooth muscle cells contributes to this accumulation, while Netrin-1 promotes vascular smooth muscle migration in atherosclerosis. In restenosis, inward remodeling of the vessel wall inhibits blood flow to surrounding tissues. Secretion of Slit-2 by vascular smooth muscle cells inhibit leukocyte recruitment and endothelial activation. Netrin-1 is released by the vascular smooth muscle, which causes endothelial nitric oxide (NO) release and promotes vascular smooth muscle quiescence. Together Slit-2 and Netrin-1 may serve as protection against neointimal hyperplasia and restenosis.

Cardiovascular disease, the leading cause of death world-wide, manifests as multiple pathologies despite common comorbidities like obesity, smoking, metabolic dysfunctions, and sedentary lifestyle (Goldberg, 2003). The most common cause of death is attributed to atherosclerosis, the accumulation of lipids, leukocytes, smooth muscle cells, necrotic cell debris, and extracellular matrix in the vascular wall of large arteries. Increasing plaque size reduces lumen diameter and blood flow to various tissues; however, thrombus formation due to plaque rupture or superficial erosion leads to clinical ischemic events characteristic of cardiovascular disease (Stary et al., 1995). While leukocytes infiltrate the developing lesion via transmigration through the endothelium, vascular smooth muscle cells undergo a phenotypic conversion and migrate into the neointima (Gerrity, 1981; Louis and Zahradka, 2010). Additionally, vascular smooth muscle has the capacity to shift to a phagocytic phenotype, where smooth muscle cells upregulate macrophage markers while smooth muscle markers are significantly downregulated. A surprising number of plaque-associated cells are in fact smooth muscle-derived, regardless of contemporary markers for other cell types (Shankman et al., 2015). Since advanced atherosclerosis is characterized by the accumulation of predominantly smooth muscle-originating cells as well as smooth muscle-derived fibrous tissue and calcium, the smooth muscle cells play a central role in the progression of the disease (**Figure 5**). While the accumulation of vascular smooth muscle cells contributes to plaque size and remodeling, they also comprise the fibrous cap, which serves as a protective barrier against plaque rupture and thrombotic complications (Newby and Zaltsman, 1999). Despite the importance of smooth muscle cells in plaque formation and progression, current therapeutics largely focus on reducing circulating plasma lipid levels with statin therapy (US Preventive Services Task Force et al., 2016). However, since the benefits of statins exhibits high patient-to-patient variability due to polymorphisms, drug interactions, and adverse side effects (Golomb and Evans, 2008; Davies et al., 2013), alternative therapeutic targets may be indicated in cardiovascular disease management. (Hackam and Anand, 2003).

A common surgical intervention with atherosclerotic stenosis is stent placement or balloon angioplasty (Ghanie, 2009). The mechanisms behind restenosis are well characterized, where damage or loss of the endothelium following intervention exposes basement membrane proteins and vascular smooth muscle to circulating blood flow. The negative effects of this are twofold: (1) platelets adhere to the extracellular matrix proteins releasing a variety of growth factors including PDGF (Bergmeier and Hynes, 2012), and (2) vascular smooth muscle cell proliferation and migration results in robust inward remodeling (**Figure 5**) (Louis and Zahradka, 2010). Although immediate restoration of blood flow provides benefit and protects damage from prolonged ischemia, the incidence of restenosis occurs with approximately 30% of patients experiencing constrictive remodeling, despite the use of drug-eluting stents (Cassese et al., 2015). Drug eluting stents reduce smooth muscle cell proliferation and migration through release of rapamycin and taxol (Dibra et al., 2005); however, these also inhibit re-endothelialization which is crucial to restore homeostasis of the vessel wall (Yurdagul et al., 2014). Furthermore, taxol-eluting stents are associated with late-stent thrombosis compared to rapamycin- or even bare-eluting stents (Stettler et al., 2007). Ultimately strategies to target smooth muscle growth while preventing endothelial inhibition are ideal to prevent restenosis and thrombosis following intervention.

EphA2 is readily detectable in multiple cell types within the atherosclerotic plaque showing high expression in the endothelium, macrophage population, and neointimal vascular smooth muscle (Funk et al., 2012; Finney et al., 2017). Intriguingly, EphA2 is undetectable medial vascular smooth muscle and quiescent smooth muscle *in vitro*, suggesting a differential expression of EphA2 with smooth muscle phenotype (Finney et al., 2017). Global deletion of EphA2 attenuates plaque size and progression in Apoe knockout mice, which may be in part mediated by vascular smooth muscle due to its observed reductions in the plaque smooth muscle cell content and corresponding loss of fibrous tissue (Finney et al., 2017). Indeed, depletion of vascular smooth muscle EphA2 *in vitro* reduces proliferation and fibronectin deposition (Finney et al., 2017). Intraplaque angiogenesis results in a premature, leaky vessel with poor pericyte coverage and is associated with weakened fibrous caps (Kolodgie et al., 2003; Michel et al., 2014). However, deletion of EphA2 does not alter intraplaque angiogenesis (Finney et al., 2017). Since depletion of EphA2 reduces the proliferative and migratory effects of vascular smooth muscle cells following scratch assay (Finney et al., 2017), inhibiting EphA2 may blunt neointimal hyperplasia and restenosis.

Cell migration by many cell types largely drives atherosclerosis. These migratory cues maintain cellular accumulation either through attractive cues into the plaque or repulsive cues which inhibit egression. Since Netrin-1 exerts chemorepulsive effects (through interactions with Unc5B) or chemoattractive effects (through interactions with either DCC or neogenin) (Keleman and Dickson, 2001), the specific contributions of Netrin-1 in atherosclerosis are of interest. While Netrin-1 gene expression is reduced in atherosclerotic plaque compared to normal thoracic arteries (Oksala et al., 2013), Netrin-1 protein is histologically detectable in regions of hypoxia in atherosclerotic plaques and colocalizes with both macrophage and smooth muscle markers (Oksala et al., 2013; Ramkhelawon et al., 2013). In addition, Netrin-1’s cognate receptor Unc5B gene expression is enhanced in atherosclerotic vessels compared with normal thoracic arteries, and is closely associated with the stable plaque phenotype (Oksala et al., 2013). Despite this, neither Unc5B nor DCC protein are highly expressed in vascular smooth muscle cells *in vitro*; however, neogenin is readily detectable (van Gils et al., 2012). Since stable plaques are characterized by the presence of a smooth muscle-rich fibrous cap (Stary et al., 1995), the observation of these guidance cues implicates a potential role for Netrin-1 and neogenin signaling in stable plaque morphology and progression. Indeed, Netrin-1 acts as a chemoattractant for vascular smooth muscle cells, which appears to be through signaling with neogenin as blockade of these interactions abrogates Netrin-1 mediated chemoattraction (van Gils et al., 2012). These effects are also observed *in vivo*, since deletion of Netrin-1 from bone marrow-derived lineages reduces smooth muscle content in atherosclerosis with associated reductions in apoptosis (van Gils et al., 2012). The association of vascular smooth muscle apoptosis and plaque vulnerability is well-established (Boyle, 1999), further conferring the detrimental role of Netrin-1 interactions with vascular smooth muscle receptors. Intriguingly, DCC or Unc5B promote apoptosis in the absence of Netrin-1, leading to the hypothesis that interactions are required to maintain cell survival in various cancer models (Kefeli et al., 2017). However, since DCC is not highly expressed in the atherosclerotic plaque (van Gils et al., 2012), it likely does not contribute to intraplaque apoptosis.

Slit2 is highly expressed in vascular smooth muscle cells following vascular injury. Since Slit2 is secreted, it may induce a paracrine effect on neighboring Robo receptors (Liu D. et al., 2016). However, Slit2 inhibits smooth muscle migration through inhibition of Rac and Cdc42 activity (Liu et al., 2006). Since balloon angioplasty additionally leads to local recruitment of inflammatory cells and generalized endothelial activation (Liu D. et al., 2016), this upregulation of Slit2 may be beneficial because Slit2 maintains endothelial barrier function (London et al., 2010) and minimizes leukocyte recruitment (Kanellis et al., 2004). Therefore, Slit–Robo signaling may be a potential therapeutic target in vascular injury.

## Guidance Molecules as Potential Therapeutic Targets

Aberrant EphB-ephrinB signaling in vascular smooth muscle affect blood pressure regulation and may be a useful target to treat hypertension. Since these alterations appear to be sex-hormone specific, drugs targeting these guidance molecules may exhibit differential effects depending on the patient’s gender. Although the only studies to date linking Ephs and ephrins with blood pressure regulation and hypertension examine EphB-ephrinB signaling (Luo et al., 2012; Wang et al., 2015c, 2016a,b,c), this does not exclude EphAs contributing to blood pressure regulation. In particular, both EphA-ephrinA and EphB-ephrinB signaling are implicated in vascular smooth muscle contractility (Deroanne et al., 2003; Ogita et al., 2003; Wu et al., 2012; Wang et al., 2015c, 2016a). EphA4 is highly expressed in vascular smooth muscle and regulates contraction through RhoA small GPTase activation (Ogita et al., 2003; Finney et al., 2017), and therefore may play a role in hypertension. Although soluble EphA receptor effectively blocks forward signaling in cancer (Dobrzanski et al., 2004), soluble EphA4 prevents vascular reinnervation following vascular denervation suggesting potential off-target effects of this therapeutic.

During atherosclerosis and restenosis, vascular smooth muscle cells exhibit the remarkable ability to change phenotypes, where they suppress their contractile markers while upregulating migratory and fibroproliferative responses (Owens et al., 2004). These smooth muscle cells, deemed a “synthetic” phenotype, contribute to plaque advancement and neointimal hyperplasia. EphA2, upregulated in synthetic vascular smooth muscle of the plaque compared to normal vessel wall, contributes to plaque progression through local proliferation, migration, and provisional matrix accumulation (Finney et al., 2017). Similarly, Netrin-1 augments smooth muscle content of the atherosclerotic plaque though interactions with smooth muscle neogenin, which enhances chemoattraction (van Gils et al., 2012). During stent angioplasty, damage to the endothelial layer promotes leukocyte recruitment, platelet aggregation, and vascular smooth muscle hyperplasia (Nakatani et al., 2003). Together these lead to the incidence of restenosis, a common complication with vascular intervention. Since vascular smooth muscle upregulates Slit2 following vascular injury and Slit2 inhibits vascular smooth muscle migration (Liu et al., 2006; Liu D. et al., 2016), Slit2 may serve as a useful therapeutic for restenosis.

Thrombus and neointimal hyperplasia are common side-effects of stent angioplasty, and patients are often provided oral anti-coagulant therapies (ten Berg et al., 2001). However, a common risk factor involves hemorrhagic complications (Shoeb and Fang, 2013). Drug-eluting stents provide a directed application of therapeutic agents to the vessel wall and often target proliferation in an attempt to blunt neointimal hyperplasia (Slavin et al., 2007). While inhibition of vascular smooth muscle is crucial in preventing neointimal hyperplasia, growth of the endothelial cells is critical for re-endothelialization and restoration of vessel wall homeostasis (Finn et al., 2007). Therefore, directed inhibition of vascular smooth muscle growth is indicated when preventing restenosis. Netrin-1, although soluble and capable of interacting with many cell types, exerts differential effects on the endothelium and vascular smooth muscle. While Netrin-1 promotes NO release from the endothelial cells (Zhang and Cai, 2010), it interacts with vascular smooth muscle DCC to inhibit migration (Liu et al., 2017). Since NO maintains vascular smooth muscle quiescence (Napoli et al., 2013), Netrin-1 may reduce vascular smooth muscle hyperplasia through multiple targets. Indeed, infusion of Netrin-1 blunts neointimal hyperplasia in a mouse wire injury model (Liu et al., 2017), suggesting therapeutic potential following stent angioplasty.

With the development of the human genome project, the identification of single nucleotide polymorphisms (SNPs) and their association with human disease has been of interest for the past two decades (Collins et al., 1997). Several guidance molecule polymorphisms predict the age of onset, susceptibility, and survival rate of Parkinson’s disease (Lin et al., 2009), and a coronary artery disease (CAD) genome-wide association study identified an association with semaphorin-plexin signaling (Ghosh et al., 2015). Furthermore, a SNP in the Neuropilin1 gene is linked with congenital heart defects (Cordell et al., 2013), and deletion of Nrp1 in vascular smooth muscle and cardiomyocytes leads to cardiac dysfunction and impaired metabolism (Wang et al., 2015b). Although multiple SNPs in EphA2 are most commonly linked with congenital cataracts (Shiels et al., 2008), the EphA2 gene locus is associated with premature myocardial infarction in humans and susceptibility to atherosclerosis in mice (Sulman et al., 1997; Wang et al., 2004). Since deletion of EphA2 reduces inflammation and fibrosis associated with atherosclerosis (Finney et al., 2017), the development of a drug targeting EphA2 is an attractive option in treating cardiovascular disease.

## Conclusion and Future Directions

The topics described in this review cover the roles of guidance molecules in vascular smooth muscle and pericyte function in development and disease. Although basic cellular responses (migration, proliferation, and attachment) are highly conserved between most guidance molecule families and cell types, the interactions between ephrins, netrins, semaphorins, and slits and their cognate receptors are highly dynamic and tightly regulated. This appears to be due to differential expression of guidance molecules, which permits both spatial and temporal regulation. For example, ephrinB2 is virtually undetectable in vascular smooth muscle early in development but is highly expressed in mature smooth muscle and pericytes (Gale et al., 2001). This delayed expression may be critical during angiogenesis, as ephrinB2 is required for pericyte coverage and attachment to the vascular endothelial layer (Foo et al., 2006). In addition, Netrins promote vascular smooth muscle migration while paradoxically inhibiting endothelial migration (Fan et al., 2008; Lejmi et al., 2008). Since vascular smooth muscle cells express neogenin and the endothelium requires both neogenin and Unc5B for signaling (Park et al., 2004; Lejmi et al., 2008), the disparate responses are likely due to alternative receptor expression.

As this review clearly indicates, the various models of physiological and pathological vessel remodeling are regulated by multiple guidance cues, often with contrasting effects. Therefore, future studies should seek to provide a more comprehensive view of guidance molecule signaling within particular pathological conditions. For example, Semaphorin and Netrin both promote pericyte migration in angiogenesis through plexin/neuropilin and neogenin, respectively (Park et al., 2004; Fan et al., 2008; Jurisic et al., 2012; Zhou et al., 2014). Therefore, potential crosstalk and compensation between these guidance cues may limit the therapeutic potential of targeting only semaphorin or netrin alone. Furthermore, since some guidance molecules are capable of reverse signaling to affect cell function in the ligand expressing cell, it is often difficult to decipher whether forward or reverse signaling is affected by blocking or deleting guidance molecules and their receptors. For example, deletion of EphA2 reduces atherosclerotic plaque size and progression in athero-prone mice (Finney et al., 2017); however, since its cognate ligand ephrinA1 is also detected in vascular smooth muscle, macrophages, and endothelial cells (Funk et al., 2012; Finney et al., 2017), it is unclear whether some of these effects aren’t due to altered reverse signaling through the ligand rather than forward signaling through the receptor. Therefore, a more comprehensive analysis of receptor and ligand signaling responses are required to determine how modulating these cues affect cell behavior.

Another major limitation in guidance molecule research is the improper presentation of ligands to their cognate receptors. Investigators often utilize soluble ligands to replicate signaling by typically cell-bound or matrix-bound guidance molecules (Salvucci et al., 2009; Wang et al., 2016a). However, these ligands may differentially regulate cell processes depending on whether they are soluble or substrate-bound. For example, soluble ephrinA1 robustly activates EphA2 at early time points, but sustained interaction promotes receptor endocytosis and lysosomal degradation in a c-Cbl-dependent manner (Walker-Daniels et al., 2002). In contrast, bound ephrinA1 on synthetic lipid membranes interacts with EphA2 and *trans*-endocytoses through ADAM10 proteolytic processing (Greene et al., 2014). Intriguingly, this uptake is dependent on free lateral movement of ephrinA1, as patterned substrate that inhibits ligand clustering prevents *trans*-endocytosis (Greene et al., 2014), suggesting Eph-ephrin signaling sensitivity to membrane dynamics. Better models of cell-bound and substrate-bound guidance molecule signaling are needed to clarify how these cues function in the context of the normal microenvironment.

While many studies inhibit guidance molecules and their receptors (e.g., genetic deletion, small interfering oligonucleotides, or blocking antibodies) and observe the resulting cell response, few examine specific signaling pathways that lead to these cellular processes. Identifying these pathways in parallel with overall cell function helps refine our understanding of how guidance molecules affect cellular machinery and provide further groundwork for therapeutic potential. Furthermore, by understanding specific signaling affected by guidance molecules, we can identify how these guidance cues integrate with other environmental signals, such as matrix composition, soluble signaling mediators, and tissue mechanics.

## Author Contributions

AF drafted, wrote, and edited the manuscript and created the figures and table. AO drafted, wrote, and edited the manuscript, and edited the figures.

## Conflict of Interest Statement

The authors declare that the research was conducted in the absence of any commercial or financial relationships that could be construed as a potential conflict of interest.
